# Diluting 2.5% Viscoadaptive Sodium Hyaluronate to Prevent Postoperative Intraocular Pressure Rise After Phacoemulsification: A Pilot Study

**DOI:** 10.7759/cureus.33499

**Published:** 2023-01-08

**Authors:** Aysha Nusef, Abdulla Almoosa, Wael Wagih

**Affiliations:** 1 Ophthalmology, King Hamad University Hospital, Busaiteen, BHR

**Keywords:** postoperative intraocular pressure, viscoelastic agent, postoperative, intraocular pressure, ophthalmic viscosurgical device, sodium hyaluronate, phacoemulsification

## Abstract

The most common complication with intraoperative viscoelastic use is an immediate elevation of intraocular pressure, particularly if the viscoelastic agent remains in the eye, which can cause serious effects. Complications may include severe ocular pain, corneal epithelial edema, and increased risk of anterior ischemic optic neuropathy and retinal artery occlusion. This study aims to find an efficient and safe adjunctive method to decrease the incidence of postoperative intraocular pressure rise. When cohesive viscoelastic agents were unavailable due to the pandemic, we diluted Microvisc 2.5% sodium hyaluronate ophthalmic viscosurgical device by 50% prior to intraocular lens implantation. Twelve eyes are included in this study, which are divided into two groups. The study followed a double-blinded methodology in which the physician and the patient were unaware of what group they were in. The first group (seven patients, seven eyes) was treated using the diluted Microvisc 2.5%, and the second group (five patients, five eyes) was controlled with the undiluted Microvisc 2.5%. The primary variable was intraocular pressure, measured at four different timeline points (baseline, day 1, week 1, and month 1). We found that the technique used had easier irrigation and aspiration with minimal viscoelastic agent left in the bag, leading to a lower postoperative intraocular pressure spike. Analysis was conducted using the Wilcoxon signed rank test, and significance was noted between the two groups on postoperative day 1 (p=0.042). The analysis also included the populations’ comorbidities (hypertension and diabetes) and postoperative outcomes (pain, corneal edema, and visual acuity).

## Introduction

Since 1970, various viscoelastic substances have been used in ocular surgeries, such as sodium hyaluronate, methylcellulose, collagen, polyacrylamide, and chondroitin sulfate [1-3]. Sodium hyaluronate is a biological polysaccharide with a high molecular mass existing in the extracellular matrix of connective tissues. It can be found in the ocular structure, including corneal endothelium, aqueous, and vitreous humor [1], and protects the corneal endothelium and maintains the anterior chamber chemistry during cataract surgery [4]. However, the adverse effect of the sodium hyaluronate is postoperative intraocular pressure (IOP) rising within 24 hours after surgery and remains a concern for most surgeons [5,6].

Viscoelastic agents have diverse viscosity, concentration, and molecular size that can affect their elimination with irrigation, aspiration, and outflow via trabecular meshwork [3,7]. IOP rise can be prevented via numerous methodologies such as using a low molecular weight viscoelastic agent, avoiding overfilling the anterior chamber with viscoelastic intraoperatively, and ensuring the removal of all viscoelastic material via irrigation and aspiration at the end of surgery [8,9]. As such, the primary concern is that viscoelastic agents can remain behind the intraocular lens (IOL) during surgery, which makes it critical to be removed, predisposing to IOP spikes [3,10,11].

Viscoelastic agents can be categorized into two groups, cohesive and dispersive, based on their rheological and physicochemical properties. Dispersive agents, unlike cohesive agents, have low molecular weights, have shorter molecular chains, and remain more in the anterior chamber. As a result, the dispersive agents, therefore, require longer aspiration time for their ample removal. Incomplete removal of viscoelastic material can cause an elevated IOP postoperatively [2,12].

Microvisc 2.5% sodium hyaluronate ophthalmic viscosurgical device (OVD) does not fit the classification since it is a viscoadaptive product aimed to adjust under the fluctuating level of turbulence. During capsulorhexis, or under low shear, it acts as cohesive and during accelerated levels of turbulence. On the other hand, during phacoemulsification, it acts as a pseudodispersive since it becomes fracturable [13].

During the COVID-19 pandemic, the supply of medication to our hospital was delayed due to the worldwide supply chain shortage. Cohesive viscoelastic agents were only available for a few months. This forced us to use the Microvisc 2.5% sodium hyaluronate, which led to the rise of postoperative IOP. A proposed solution for the increased IOP by the surgeon was diluting the Microvisc 2.5% sodium hyaluronate OVD to 1.25% to potentially decrease the anterior chamber overfilling, allow easier viscoelastic material removal via irrigation and aspiration, and decrease the remaining viscoelastic material behind the IOL.

## Materials and methods

The Institutional Review Board approved the study at King Hamad University Hospital (IRB# 22-520). All patients who presented to King Hamad University Hospital in the Kingdom of Bahrain undergoing phacoemulsification and who consented were eligible for inclusion in this study. The exclusion criteria for this study were patients with a history of glaucoma, patients with elevated preoperative IOP (defined as IOP higher than 21 mmHg), or patients unwilling to sign informed consent.

The informed consent process was done physically and patient signature was taken. The patient was informed about the purpose of the study, the possible risks or discomfort, and the possible benefits, and that no financial considerations were in place. Additionally, patients were given the opportunity to ask the doctor to explain any words or information that they do not clearly understand. Voluntary consent measures and confidentiality clauses were included in the form.

The patients were segregated into two groups: treatment and control. The sodium hyaluronate Microvisc 2.5% used intraoperatively was diluted to 50% using a balanced salt solution (BSS) before IOL implantation that was performed for the treatment group. On the other hand, the undiluted Microvisc 2.5% was used in the control group. The OVD dilution was done using the Microvisc 2.5% syringe, and after adding the BSS, the OVD syringe plunger was pulled back and forth until the viscoelastic agent was diluted.

The study recruited patients based on the convenience sampling technique following a randomized control trial design. This study followed a double-blinded design. The patient and the consultant physician performing the surgery were unaware if they were given the diluted Microvisc 2.5% or the undiluted Microvisc 2.5%. Patients were randomly assigned into two groups by simple randomization, and the gatekeeper of the study recorded the information as necessary and kept it in a blind log. One consultant physician performed all surgeries.

Preoperatively, all patients were given acetazolamide tablets 250 mg orally and dilated with Mydriacyl 1% and phenylephrine 10% as their standard preoperative preparation is done before cataract surgery. Baseline IOP was taken in the clinic prior to surgery.

As for the technique, we used the Microvisc 2.5% syringe (0.85 mL) and equally filled it with sterile BSS. The syringe plunger was pulled back and forth until the Microvisc 2.5% was diluted for five times. This process was done in the preloaded Microvisc 2.5% syringe by a trained scrub nurse immediately before injecting the solution in the eye to implant the IOL. The physician performing the surgery was unaware of the type of OVD given, whether diluted or undiluted, which ensured that the irrigation and aspiration time was not affected by knowing the type of OVD given. The diluted OVD was injected prior to lens implantation only.

Postoperatively, patients were asked to visit the ophthalmology clinic on day 1 for IOP measurement, using Goldmann applanation tonometry and routine eye examination. Patients were asked to visit the ophthalmology clinic for re-evaluation of the IOP during their routine postoperative follow-up visits one week after surgery and one month after surgery, as well as if needed.

There were 13 eyes (13 participants) that consented to participate in the study. Patients were examined on the first postoperative day, after one week, and after one month. One patient was excluded due to the availability of follow-up data, resulting in a sample size of 12 eyes (12 participants). The participants were randomly categorized into two groups: treatment and control. Those in the control group received the undiluted Microvisc 2.5%, and those in the treatment group received diluted Microvisc 2.5%. The same surgeon performed all surgeries and was blinded to which group the patient was in.

## Results

Data collected were anonymized by removal of patient identification variables and then analyzed using SPSS Version 25 (IBM Corp., Armonk, NY). A p-value of less than 0.05 was considered significant. There were seven (58.3%) male patients and five (41.7%) female patients. In addition, hypertension and diabetes, considered significant comorbidities, were looked for. There were seven (58.3%) hypertensive patients and five (41.7%) non-hypertensive patients. On the other hand, the population was split in half with a diabetes diagnosis.

There were a total of seven eyes in the treatment group. The average age for the treatment group was 62.28 years, with a minimum age of 30 years and a maximum age of 77 years. On the other hand, there were five eyes in the control group. The average age was 63.6 years, with a minimum age of 49 years and a maximum age of 74 years. Regression analysis was conducted to identify which factors matter most in the two groups and which can influence each other (Table 1).

**Table 1:** Demographics of the population

	Treatment (n=7)	Control (n=5)	Total (n=12)	P-value
Age (years)	Average: 62.28	Average: 63.60	Average: 62.83	0.670
	Minimum: 30	Minimum: 49	Minimum: 30	
	Maximum: 77	Maximum: 74	Maximum: 77	
Gender	Male: 4 (57.2%)	Male: 3 (60%)	Male: 7 (58.3%)	0.920
	Female: 3 (42.8%)	Female: 2 (40%)	Female: 5 (41.7%)	
Hypertension	Yes: 4 (57.2%)	Yes: 3 (60%)	Yes: 7 (58.3%)	0.535
	No: 3 (42.8%)	No: 2 (40%)	No: 5 (41.7%)	
Diabetes	Yes: 3 (42.8%)	Yes: 3 (60%)	Yes: 6 (50%)	0.877
	No: 4 (57.2%)	No: 2 (40%)	No: 6 (50%)	

**Figure 1:**
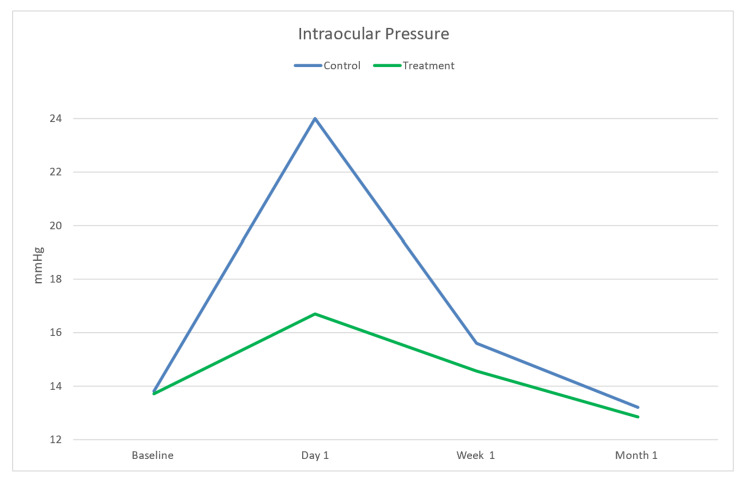
Changes in mean intraocular pressure between both groups across the four time points.

IOP was measured at four different timeline points (baseline, day 1, week 1, and month 1), and analysis was conducted using the Wilcoxon signed rank test. The Wilcoxon signed rank test was used to compare different data points for the same group, while a Mann-Whitney test was used to compare the two groups at a single point. There was no significance during the baseline point (p=0.785) and at the one-month point (p=0.414); additionally, there was weak statistical evidence and a lack of significance during the one-week point (p=1.000). On the other hand, there was strong evidence and significance at the one-day point (p=0.042). Descriptive statistics were also performed for the IOP measurements (Table 2). Additionally, IOP was illustrated to showcase the difference in means between both groups (Figure 1).

**Table 2:** IOP descriptive and mean statistics *Significant p-value IOP, intraocular pressure

Time points		Maximum IOP	Minimum IOP	Mean IOP	SD	Wilcoxon signed rank test
Baseline	Treatment	17	10	13.71	2.49	0.785
	Control	19	10	13.80	3.34	
1 day	Treatment	27	13	16.71	4.75	0.042*
	Control	37	14	24.00	8.36	
1 week	Treatment	16	14	14.57	0.78	1.000
	Control	22	14	15.60	3.57	
1 month	Treatment	11	16	12.85	1.86	0.414
	Control	11	18	13.20	2.94	

Furthermore, postoperative outcomes were reported. Pain was self-reported in 16.7% of all cases, and corneal edema was reported in 41.7% of all cases. Regression analysis and frequencies were computed (Table 3).

**Table 3:** Postoperative outcomes

	Treatment (n=7)	Control (n=5)	Total (n=12)	P-value
Pain	Yes: 0 (0%)	Yes: 2 (40%)	Yes: 2 (16.7%)	0.164
	No: 7 (100%)	No: 3 (60%)	No: 10 (83.3%)	
Corneal edema	Yes: 2 (28.5%)	Yes: 3 (60%)	Yes: 5 (41.7%)	0.889
	No: 5 71.4%)	No: 2 (40%)	No: 7 (58.3%)	

Visual acuity was also recorded. The scores were translated to measure the logarithm of the minimum angle of resolution (logMAR) per group and as a total population. This was computed and categorized into four groups: (1) mild or no visual impairment: presenting visual acuity (PVA)≤0.5 logMAR (20/63); (2) moderate visual impairment: 0.5 logMAR (20/63)<PVA≤1.0 logMAR (20/200); (3) severe visual impairment: 1.0 logMAR (20/200)<PVA≤1.3 logMAR (20/400); and (4) blindness: PVA>1.3 logMAR (20/400) [14]. It should be noted that one patient was not included in the visual acuity analysis as it was reported as a counting fingers. A Mann-Whitney test was used, which showed no significance in the means between the two groups (p=0.537).

## Discussion

OVDs have been used in phacoemulsification since 1972, and they played a huge role in eye protection during cataract surgery [1,10,12,15]. Cohesive OVDs are considered ideal when implanting a lens during phacoemulsification because they can be removed faster than dispersive OVDs [10]. Viscoadaptive OVD is considered a new category - a high-viscosity OVD that has the characteristics of changing into cohesive and adaptive depending on the surgical setting [2]. Total removal of the viscoelastic device is a critical step post-IOL insertion in phacoemulsification. If OVD stays in the anterior chamber or behind the IOL, it can cause complications, including a rise in IOP, pain, and corneal endothelial loss [8]. Implantation of IOL with OVD dilution was never reported in the literature.

In our population, we found some patients with hypertension and diabetes, but it was not found to be significant. With evidence-based medicine being a major factor in our current practice, any risk from associated hypertension, such as acute intra-operative suprachoroidal hemorrhage, is likely to be of minimal significance [16]. In our study, we found no complications from associated hypertension. However, one patient reported pain postoperatively, and four patients reported corneal edema. On the other hand, in the diabetes population, two patients reported pain, and four patients reported corneal edema postoperatively. Since diabetes is a significant concern in the Kingdom of Bahrain, we emphasize the importance of its presence in our population. However, patients with diabetes were treated similar to other patients in this study. Following evidence-based practice and the advancements in ophthalmology research, Sekelj et al. reported no difference in the outcomes between non-diabetic and those with type 2 diabetes after phacoemulsification [17].

Painless, fast, and uncomplicated cataract surgery is the most crucial outcome of phacoemulsification. In this study, our primary measure was IOP to suggest a difference between the two groups. On postoperative day 1, one patient from the undiluted group was recorded to have an IOP of more than 30 (37 mmHg), unlike the diluted group in which no patient was recorded to have an IOP above 30 mmHg. In correlation with the IOP, the diluted group did not report any pain on the first day postoperative. On the other hand, pain was reported in 40% of the undiluted group. Otherwise, IOP measurements were insignificant at the other time points (baseline, week 1, and month 1). Figure 1 suggests that although the IOP measurements at baseline were similar, the IOP post-surgery for the control group was always higher than the treatment. Moreover, postoperative outcomes such as pain, corneal edema, and visual acuity were not significant in this sample, which might have significance if the study was repeated with a larger sample size.

Significance was seen in the postoperative IOP measurement on day 1 through descriptive analysis, where the mean IOP for the diluted sample was 16.71 mmHg. Meanwhile, the mean IOP for the undiluted sample was 24 mmHg. The Wilcoxon signed rank test proved that there was statistical significance as the p-value is 0.042. The extent of the rise in IOP on postoperative day 1 is linked to the viscosity of the OVD. Viscoadaptive OVD has a higher viscosity than cohesive and adaptive OVDs, which causes incidents of higher IOP spikes [2]. Hence, surgeons must know these characteristics when dealing with these new-generation viscoelastic agents.

The technique that was used in this research, up to our knowledge, is the first reported in the literature. Throughout the study, we noticed the strengths that our technique portrayed, as seen from the results. The strengths not only lowered the incident of raised IOP and ocular pain from postoperative day 1 but also initiated faster irrigation and aspiration time with less viscoadaptive remaining in the anterior chamber, and rotation and repositioning of IOL in the bag was not an issue. Cost-effectiveness is also one of these advantages when cohesive OVDs are not available and allows the surgeon to use one type of viscoelastic agent.

The weakness or limitation of this study was that the sample size collected was not strong enough to deliver highly significant results. Due to the blinded research methodology, the study was limited to only one physician to maintain accurate data records, which led to a smaller sample size. The study could have also benefited from adding more variables that supported the population's outcome variables, including anterior chamber reaction or risk of endophthalmitis. In addition, patients with a higher risk of complications were not included in this study, for example, patients with pseudoexfoliation or floppy iris syndrome. Also, due to the nature of the self-administration of postoperative medication, patients may have not adhered to the requested postoperative regimen.

Furthermore, since the viscoadaptive OVDs have been reported to stay behind the IOL [2], we aimed to reduce the viscoelastic material captured behind the artificial lens and in the trabecular meshwork through our technique using Microvisc 2.5%. The dilution technique that was done by the trained scrub nurse was faced with a few limitations. In some cases, the diluted OVD had a clumpy appearance as reported by the nurse, and could have decreased the visibility and increased the risk of posterior capsule injury during IOL implantation. However, there was no report of posterior capsule rent in our sample. On the other hand, the clumpy appearance of viscoelastic material facilitated easier irrigation and aspiration compared to the undiluted viscoelastic material.

## Conclusions

The aim of diluting Microvisc 2.5 % sodium hyaluronate was to have easier irrigation and aspiration with a minimal viscoelastic agent left in the bag, leading to lower postoperative IOP spike and minimal postoperative pain. After diluting the viscoadaptive OVD with BSS, IOP spikes and ocular pain decreased. There was a clinically significant difference between the diluted and undiluted viscoelastic group on postoperative day 1. The diluted viscoelastic group showed a lower incidence of IOP spikes and no ocular pain postoperative. This hypothesis should be studied in future research as these results are conducted using a small sample size.

In the future, we recommend that researchers continue innovating new techniques that will help surgeons adapt to the new generation of OVDs. Also, take advantage of their positive impact during cataract surgery and reduce the limitation of IOP spikes and ocular pain on postoperative day 1. Diluting the new generation of viscoadaptive OVDs is one of these innovations that can be cost-effective, reduce the time of surgery, and decrease IOP spikes.
